# The association between health-related quality of life and problem gambling severity: a cross-sectional analysis of the Health Survey for England

**DOI:** 10.1186/s12889-024-17816-3

**Published:** 2024-02-12

**Authors:** Esther Moore, Robert Pryce, Hazel Squires, Elizabeth Goyder

**Affiliations:** School of Medicine and Population Health, University of Sheffield, Sheffield, UK

**Keywords:** Gambling harms, Gambling problems, Health utility, EQ-5D, Problem Gambling Severity Index

## Abstract

**Background:**

Problem gambling can lead to health-related harms, such as poor mental health and suicide. In the UK there is interest in introducing guidance around effective and cost-effective interventions to prevent harm from gambling. There are no estimates of the health state utilities associated with problem gambling severity from the general population in the UK. These are required to determine the cost-effectiveness of interventions. This study aims to use an indirect elicitation method to estimate health state utilities, using the EQ-5D, for various levels of problem gambling and gambling-related harm.

**Methods:**

We used the Health Survey for England to estimate EQ-5D-derived health state utilities associated with the different categories of the Problem Gambling Severity Index (PGSI), PGSI score and a 7-item PGSI-derived harms variable. Propensity score matching was used to create a matched dataset with respect to risk factors for problem gambling and regression models were used to estimate the EQ-5D-derived utility score and the EQ-5D domain score whilst controlling for key comorbidities. Further exploratory analysis was performed to look at the relationship between problem gambling and the individual domains of the EQ-5D.

**Results:**

We did not find any significant attributable decrements to health state utility for any of the PGSI variables (categories, score and 7-item PGSI derived harms variable) when key comorbidities were controlled for. However, we did find a significant association between the 7-item PGSI derived harms variable and having a higher score (worse health) in the anxiety/depression domain of the EQ-5D, when comorbidities were controlled for.

**Conclusions:**

This study found no significant association between problem gambling severity and HRQoL measured by the EQ-5D when controlling for comorbidities. There might be several reasons for this including that this might reflect the true relationship between problem gambling and HRQoL, the sample size in this study was insufficient to detect a significant association, the PGSI is insufficient for measuring gambling harm, or the EQ-5D is not sensitive enough to detect the changes in HRQoL caused by gambling. Further research into each of these possibilities is needed to understand more about the relationship between problem gambling severity and HRQoL.

**Supplementary Information:**

The online version contains supplementary material available at 10.1186/s12889-024-17816-3.

## Introduction

Problem gambling, and the associated gambling-related harm, is a public health issue [1, 2]. A recent report estimated that gambling-related harm in England costs the government £412.9 million [3]. Consequently, there is interest in developing guidelines and interventions to address problem gambling and the associated harms [4, 5]. In 2019 the NHS set out its ambition to open new clinics to treat gambling addiction and since then has increased the number of clinics from one national clinic to seven clinics throughout England [6, 7]. Furthermore, the National Institute for Health and Care Excellence (NICE) is currently developing guidelines for harmful gambling [4]. When making decisions about interventions, NICE and other organisations, consider the cost-effectiveness of the intervention [8].

Cost-effectiveness can be calculated by estimating the cost per quality-adjusted life year (QALY) [9]. To estimate QALYs, a health-related quality of life (HRQoL) instrument is used to generate a profile of a condition. This profile is then valued by members of the public to get the health state utility. This process can be done either directly or indirectly [10]. Direct elicitation involves participants evaluating vignettes that describe the health condition. Indirect methods involve matching the health condition to a health profile which is generated using instruments such as the EuroQol-5D (EQ-5D) and The Short Form (36) Health Survey (SF-36), which is then mapped to utilities using value sets [11].

Both direct and indirect methods have been used to estimate a health utility for low-risk, moderate-risk and problem gambling, defined using the Problem Gambling Severity Index (PGSI) [12–15]. The PGSI consists of nine items (e.g., Item 1: Have you bet more than you could really afford to lose?) and each item is measured on a 4-point scale (from never = 0 to always = 3) [16]. Please see Additional File 1 for all the PGSI questions. The scores for each of the nine items are summed together to get a PGSI score. This score can be used to assign risk categories. A PGSI score of 0 indicates a non-problem gambler, a score of 1–2 indicates a low-risk gambler, a score of 3–7 indicates a moderate-risk gambler and a score of 8 or above indicates a high-risk gambler.

Five studies have attempted to estimate the association between problem gambling and health-related quality of life worldwide [12–15, 17]. Direct methods were used in two studies, one in Australia and one in New Zealand [12, 13]. The studies estimated that problem gambling was associated with a reduction in health state utility of between 0.44–0.54, for moderate risk a reduction of between 0.29–0.37 and low risk a reduction of 0.13–0.18 compared to no risk/ non-gamblers gamblers. Two Australian studies used the SF-36 to indirectly estimate health state utilities associated with problem gambling and estimated much smaller reductions in health state utility due to gambling [14, 15]. Problem gambling was associated with a reduction of between 0.099–0.181, for moderate risk a reduction of between 0.051–0.057 and low risk a reduction of 0.005–0.030 compared to no-risk gamblers/non-gamblers. There has only been one study conducted in the UK that has estimated utility values for varying severities of problem gambling, and this was using the EQ-5D and a population of military veterans [17]. This study reported that those experiencing problem gambling had a higher utility than those without any problems or those at moderate and low risk. Since this study only looked at the veteran population the results are not generalizable to the general population in the UK and may not be appropriate for use within a cost-effectiveness analysis of the broader population.

Cost-effectiveness analysis of treatments being evaluated by NICE in the UK, requires the EQ-5D to be used to generate utilities unless there is empirical qualitative evidence on the lack of validity of the EQ-5D showing that key dimensions of health are missing [18]. The EQ-5D is a generic measure of HRQoL with five dimensions: mobility, self-care, usual activity, pain/discomfort and anxiety/depression [19]. Each dimension has five levels: no problems, slight problems, moderate problems, severe problems and extreme problems. The responses to these questions create a health profile for each participant, which is then valued using a value set to generate the utility associated with the health condition [20]. There are concerns that it might not be appropriate to use the EQ-5D to measure gambling-related harm [12]. EQ-5D tends to focus on biological and physical health with only one domain focused on mental health and no domain that considers the impact on relationships, yet both of these are common gambling-related harms [19, 21]. Despite these concerns, there is not enough evidence to discount the use of the EQ-5D to derive utilities associated with problem gambling and a 2019 review found two studies which used the EQ-5D to measure HRQoL changes due to gambling [22–24]. It is of interest to investigate the relationship between problem gambling severity and health state utilities measured using the EQ-5D, as there is potential for these to be used in the evaluation of gambling interventions in the UK.

This study aims to use an indirect elicitation method to estimate health state utilities, using the EQ-5D, for various levels of problem gambling and gambling-related harm. This is the first study to use a nationally representative survey to estimate health state utilities associated with gambling for the general population of England.

## Method

The methods used in the analyses were based on the framework outlined by Browne et al. and a previous paper which implemented this framework [15, 25]. Following this framework, the sample was split into two groups, those who had been affected by gambling (with a PGSI score of over 1) and those who had not (with a PGSI score of 0). Key risk factors for experiencing gambling-related harm were then identified and a propensity score model was used to match participants, generating a matched sample. Key comorbidities were identified and controlled for in regression analyses used to estimate the EQ-5D-5L-derived utility values associated with varying severities of problem gambling and gambling-related harm.

### Dataset and sample

The 2018 Health Survey for England (HSE) is the only dataset in England to include data on both HRQoL, measured using the EQ-5D-5L, and gambling behaviour [26]. The HSE is a yearly cross-sectional survey of adults (defined in the HSE as over 16 years old) and children living in private households representative of the population of England. In 2018 questions regarding gambling behaviour were asked to a sample of the survey population. These included all nine questions in the PGSI, the results of which were summed to give an overall PGSI score. In 2018 questions were also asked regarding HRQoL, using the EQ-5D-5L. Of the 6923 people who had a value for PGSI score, 113 were missing a value for any domain of the EQ-5D-5L and so were excluded from the sample. This resulted in an initial sample of 6810.

### Measures

The PGSI (both the score and the categories) were used to quantify the severity of problem gambling (see Additional File 1 for the PGSI questions and scoring). The PGSI is not designed to measure gambling harm, despite it being used in this way [27]. Other studies have separated the PGSI items considering only the items that refer to the negative consequences of gambling and used the sum of these as a proxy for gambling harm. A recent study used seven out of the nine items on the PGSI as a proxy for gambling harm [28]. These seven items specifically ask about the consequences of gambling whereas two of the PGSI items asks about gambling behaviour (item 2 and 3). Therefore, we used a derived variable, the sum of the seven items related to the consequences (harms) of gambling, to determine the relationship between gambling harm and health state utility.

The HSE uses the EQ-5D-5L version of the EQ-5D to measure HRQoL [26]. Currently there is no value set for the EQ-5D-5L for the English population recommended by NICE [29]. Therefore the health profiles were mapped to the 3L version of the EQ-5D and valued using the NICE Decision Support Unit method to get the EQ-5D score which was included as one of the dependent variables [30]. For the analysis of the EQ-5D domains, the domain score was the dependent variable. Each dimension has five levels: no problems = 1, slight problems = 2, moderate problems = 3, severe problems = 4 and extreme problems = 5 so the domain scores range from 1–5.

### Statistical analysis

The first stage was to calculate the weights required for the propensity score matching. This was done using a logistic regression model to determine the likelihood of a participant having a PGSI score of 0 or a PGSI score of 1 or more. Key risk factors were considered for inclusion in the propensity model, based on those suggested by Browne et al. 2020 and the available data in the HSE [25, 26]. The chosen risk factors were: age, sex, ethnicity, National Statistics Socio-economic classification, highest qualification achieved, rural/urban residential location, marital status, presence of condition affecting behaviour (i.e., autism, attention deficit disorder or Asperger's syndrome) and household income. Table 1 shows the differences in these risk factors across the two groups. PGSI 1 + group had a higher percentage of men in the sample compared to those with a PGSI of 0, 74% compared to 43%. The PGSI 1 + group was younger, with 25% of the sample aged 16 to 29 years old compared to 15% in the PGSI 0 group. PGSI 1 + had a higher percentage of individuals of white and mixed ethnicity compared to PGSI 0, and a small proportion of the sample PGSI 1 + belonged to the Asian ethnicity. The PGSI 1 + group were generally not as educated as the PGSI 0 group, with 20% having a degree or equivalent compared to 30% in the PGSI 0 group. In terms of marital status, the PGSI 1 + group had a higher percentage of single people and cohabitees, than the PGSI 0 group. The income and occupation levels of both groups were generally similar. A higher percentage of those in the PGSI 1 + group live in urban places, compared to the PGSI 0 group. The PGSI 1 + group had a higher percentage of individuals with a long-lasting condition which affected them socially or affected their behaviour, 5% compared to 2% for the PGSI 0 group. The *P* values in Table 1 are a result of a chi-squared test, with a *P* value < 0.5 indicating that there is a statically significant difference between the PGSI 1 + and PGSI 0 groups in terms of the relevant variable. The differences in the demographics show the importance of using propensity score matching to have a sample which is balanced across key risk factors for problems with gambling.

**Table 1:** Demographic characteristics of the sample before propensity score matching

	**PGSI 0 N(%)**	**PGSI 1 + N(%)**	***P* Value**
N	6574 (97%)	236 (3%)	
Sex			< 0.001
Male	2859 (43.49%)	175 (74.15%)	
Female	3715 (56.51%)	61 (25.85%)	
Age (years)			< 0.001
16–29	960 (14.60%)	60 (25.42%)	
30–44	1575 (23.96%)	90 (38.14%)	
45–59	1733 (26.36%)	57 (24.15%)	
60–74	1628 (24.76%)	22 (9.32%)	
75 +	678 (10.31%)	7 (2.97%)	
Ethnicity			0.02
White	5750 (87.56%)	211 (89.41%)	
Black	196 (2.98%)	6 (2.54%)	
Asian	464 (7.07%)	9 (3.81%)	
Mixed	100 (1.52%)	9 (3.81%)	
Other	57 (0.87%)	1 (0.42%)	
Missing	7	0	
Highest educational qualification			0.001
NVQ4/NVQ5/Degree or equiv	1939 (29.58%)	47 (19.92%)	
Higher ed below degree	768 (11.72%)	31 (13.14%)	
NVQ3/GCE A Level equiv	1053 (16.06%)	61 (25.85%)	
NVQ2/GCE O Level equiv	1264 (19.28%)	48 (20.34%)	
NVQ1/CSE other grade equiv	224 (3.42%)	8 (3.39%)	
Foreign/other	66 (1.01%)	1 (0.42%)	
No qualification	1241 (18.93%)	40 (16.95%)	
Missing	19	0	
NS-SEC (occupation)			0.152
Higher managerial and professional occupations	846 (13.09%)	21 (8.94%)	
Lower managerial and professional occupations	1616 (25.00%)	50 (21.28%)	
Intermediate occupations	924 (14.29%)	34 (14.47%)	
Small employers and own account workers	615 (9.51%)	25 (10.64%)	
Lower supervisory and technical occupations	394 (6.10%)	18 (7.66%)	
Semi-routine occupations	1069 (16.54%)	39 (16.60%)	
Routine occupations	744 (11.51%)	38 (16.17%)	
Never worked and long-term unemployed	110 (1.70%)	2 (0.85%)	
Other	146 (2.26%)	8 (3.40%)	
Missing	110	1	
Marital status			< 0.001
Single	1235 (18.79%)	79 (33.47%)	
Married, including civil partnership	3530 (53.70%)	83 (35.17%)	
Separated, including from civil partnership	126 (1.92%)	4 (1.69%)	
Divorced, including dissolved civil partnership	449 (6.83%)	16 (6.78%)	
Widowed, including civil partnership	405 (6.16%)	6 (2.54%)	
Cohabitees	829 (12.61%)	48 (20.34%)	
Income (Equivalised Income Quintiles)			0.27
Highest Quintile (> £52,817)	1045 (19.19%)	46 (22.55%)	
Second highest Quintile (> £31,967 < = £52,817)	1077 (19.78%)	32 (15.69%)	
Middle Quintile (> £23,084 < = £31,967)	1019 (18.71%)	31 (15.20%)	
Second lowest Quintile (> £14,918 < = £23,084)	1168 (21.45%)	46 (22.55%)	
Lowest Quintile (< = £14,918)	1137 (20.88%)	49 (24.02%)	
Missing	1128	32	
Residence			0.005
Rural	1373 (20.89%)	31 (13.14%)	
Urban	5201 (79.11%)	205 (86.86%)	
Long lasting condition affecting behaviour and social skills			0.023
Yes	158 (5.31%)	11 (11.11%)	
No	2818 (94.69%)	88 (88.89%)	
Missing	3598	137	

The specification of the propensity model was decided upon using backward stepwise elimination using the Akaike Information Criteria (AIC). Age, sex, ethnicity, occupation, highest education qualification, marital status, income, residence (rural vs urban) and the presence of a long-lasting condition affecting behaviour and social skills were all considered for inclusion in the propensity model. Based on the AIC, age, highest qualification achieved and sex were included in the propensity model to calculate the likelihood of a participant experiencing a PGSI 1 + . Complete data is required for propensity score matching so those with missing values for highest qualification were removed from the sample prior to the matching (*n*= 19). There were no missing values for age or sex. Once the propensity score matching model was specified, nearest-neighbour matching was used to match participants, and the covariates' balance was checked to ensure the matching was appropriate [10]. See Additional File 2 for a Table 1.1 of the demographics of the sample after matching.

Regression models were used to predict the EQ-5D-derived utility score and each of the EQ-5D domain scores (mobility, self-care, usual activities, pain, anxiety/ depression) using the PGSI score, the PGSI categories and the 7-item derived PGSI harm variable (each in separate models). The models were run both without and without controlling for key comorbidities. The comorbidities included were chosen using the framework by Browne et al. 2020 and considering the availability of the data in HSE [25, 26]. The comorbidities included were: the presence of a long-term mental disorder, receiving disability allowance, alcohol consumption and cigarette smoking. Age, sex and ethnicity were also included in the models. This regression model is shown below in Eq. 1, with a description of each variable included in Table 2. Interaction terms were not included due to the relatively small sample from the HSE.

**Table 2:** A description of the variables included in the regression models

Variable name	Description
EQ-5D variable	Both EQ-5D utility score and EQ-5D domain scores were chosen as dependent variables
*EQ-5D score*	EQ-5D utility score and EQ-5D domain score were included as the dependent variables
*EQ-5D domain scores*	The score for each of the EQ-5D domains: mobility, self-care, usual activities, pain, anxiety/ depression, scores range from 1–5
PGSI variables	PGSI score, PGSI category and a 7-item derived harms variable were each included, separately, in the models i.e., the model was repeated with either PGSI score, PGSI category or 7-item derived harms variable as an independent variable (3 models for each dependent variable)
*PGSI score*	The total score from the PGSI, ranging from 0–27
*PGSI category*	Four categories based on the total PGSI score of the respondent 0 = No-risk/non-gamblers 1–2 = Low-risk gambler 3–7 = Moderate-risk gambler 8 + = High-risk gambler
*7-item derived harms variable*	A derived variable using 7 out of the 9 items of the PGSI (item 2 and 3 excluded), ranging from 0–21
Mental Disorder	A binary variable indicating the presence of a mental disorder as a long-lasting illness
Disability	A binary variable indicating whether the participant received any disability benefits
Alcohol	A categorical variable referring to the frequency of alcohol drunk in the past 12 months with 8 levels (1. Almost every day, 2. Five or six days a week, .3 Three or four days a week, 4. Once or twice a week, 5. Once or twice a month, 6. Once every couple of months, 7. Once or twice a year, 8. Not at all in the last 12 months/Non-drinker)
Smoke status	A categorical variable referring to cigarette smoking status (10 or more cigarettes daily, less than 10 cigarettes daily, or a non-smoker)
Age	A categorical variable referring to the age of the participant in approximately 3/5-year bands, from 16–19 years to 90 + years
Sex	A binary variable indicating male or female
Ethnicity	A categorical variable referring to the ethnicity of the participant with 5 categories: White, Black, Asian, Mixed and Other

1 $$\text{EQ}-5\mathrm D=\mathrm\alpha+\mathrm\beta_1\;\mathrm{PGSI}+\mathrm\beta_2\;\mathrm{MENTALDISORDER}+\mathrm\beta_3\mathrm{DISABILITY}+\mathrm\beta_4\mathrm{ALCOHOL}+\mathrm\beta_5\mathrm{SMOKESTATUS}+\mathrm\beta_6\mathrm{AGE}+\mathrm\beta_7\mathrm{SEX}+\mathrm\beta_8\mathrm{ETHNICITY}$$

This model was repeated for each PGSI variable (PGSI score, PGSI category and 7-item derived PGSI harm variable) and rerun both without and including the covariates described above.

## Results

**Fig. 1:**
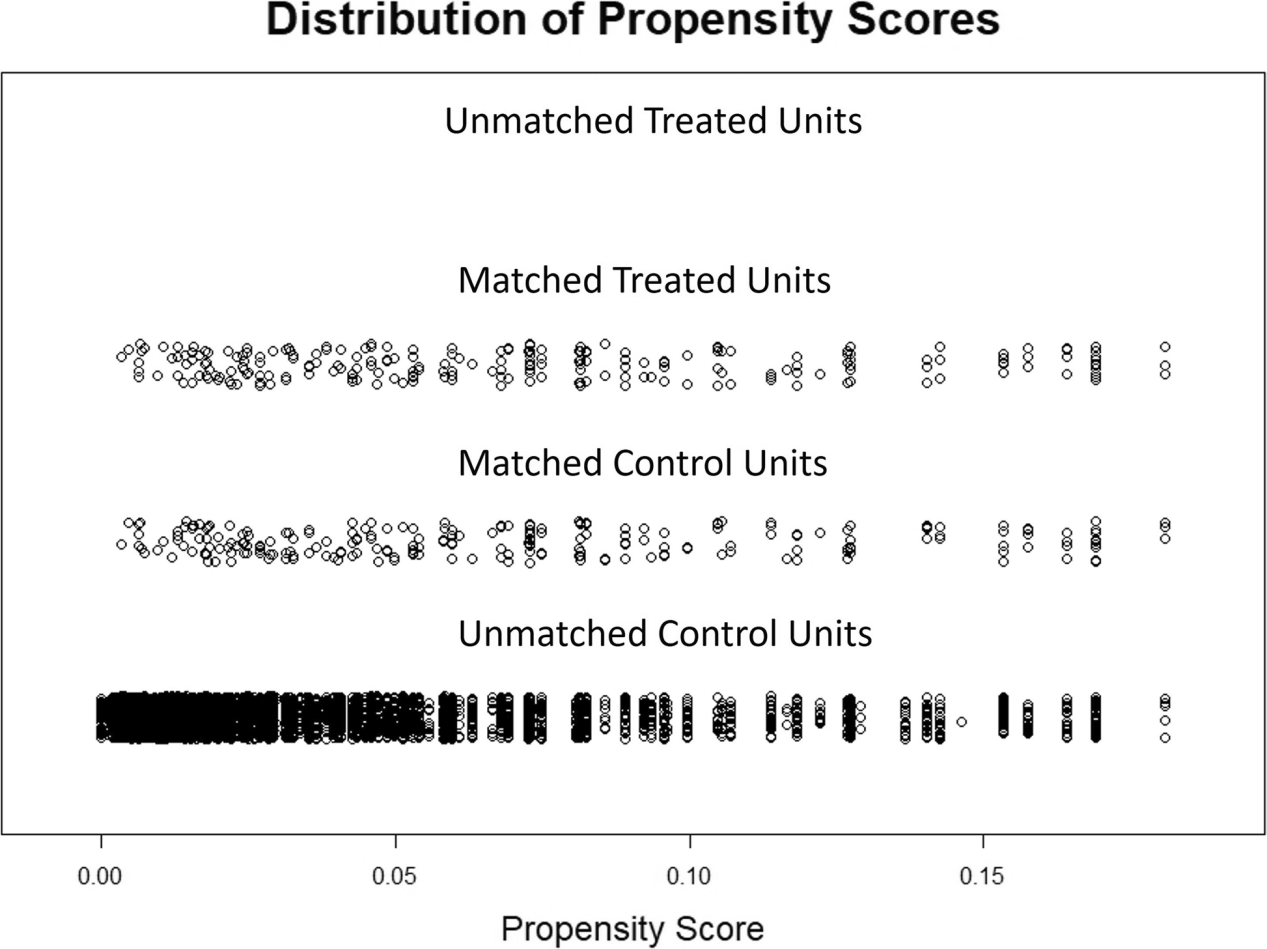
The distribution of propensity scores, before and after matching

Figure 1 shows a good overlap of propensity scores between the two groups and the sample was considered adequately matched. The regression models with no covariates included were undertaken on this matched sample of 472 participants. Thirty participants had incomplete data for the comorbidities included in some of the models, so these models were undertaken on a sample of 442 which had complete data. The mean EQ-5D score across the whole matched sample was 0.82.

Table 3 shows the mean EQ-5D score and the balance of the covariates across the PGSI categories for the sample. The *P* values indicate that only the frequency of alcohol consumption differs between the PGSI categories to a statistically significant level. However, the relationship between the frequency of alcohol drank and the PGSI category is not clear. The no-risk/non-gambler group and the high-risk category have a similar percentage of people who do not drink at all, 18% compared to 17%. Similarly, at the most frequent alcohol drinking level, 9% of no-risk/non-gamblers drink almost daily, compared to 13% of the high-risk PGSI category. All of these covariates in Table 3 were included in the regression models.

**Table 3:** The balance of the comorbidities between the PGSI categories, after the propensity score matching

	**No-risk/ Non-gamblers N (%)**	**Low-Risk N (%)**	**Moderate-Risk N (%)**	**High-Risk N (%)**	*P* value*
Total	236 (50%)	162 (34%)	51 (11%)	23 (5%)	
Mean EQ-5D score	0.83	0.82	0.77	0.74	0.136
*Frequency of alcohol intake in the past 12 months*					0.004
Almost every day	20 (9%)	10 (6%)	7 (14%)	3 (13%)	
Five or six days a week	9 (4%)	11 (6.79%)	3 (6%)	2 (9%)	
Three or four days a week	35 (15%)	28 (17%)	5 (10%)	0 (0.00%)	
Once or twice a week	56 (24%)	54 (33%)	20 (40%)	9 (39%)	
Once or twice a month	38 (16%)	23 (14%)	11 (22%)	4 (17%)	
Once every couple of months	16 (7%)	15 (9%)	1 (2%)	1 (4%)	
Once or twice a year	18 (8%)	13 (8%)	2 (4%)	0 (0%)	
Not at all in the last 12 months/Non-drinker	42 (18%)	8 (5%)	1 (2%)	4 (17%)	
Missing	2	0	1	0	
*Long-term mental health disorder*					0.149
No	207 (88%)	143 (88%)	49 (96%)	18 (78%)	
Yes	29 (12%)	19 (12%)	2 (4%)	5 (22%)	
Missing	0	0	0	0	
*Cigarettes smoked per day*					0.228
> = 10	22 (9%)	24 (15%)	8 (16%)	6 (26%)	
< 10	30 (13%)	19 (11.73%)	6 (12%)	4 (17%)	
Non-smoker	182 (78%)	119 (73%)	36 (72%)	13 (57%)	
Missing	2	0	1	0	
*Receives disability allowance*					0.241
No	191 (87%)	144 (92.31%)	42 (86%)	20 (95%)	
Yes	29 (13%)	12 (7.69%)	7 (14%)	1 (5%)	
Missing	16	6	2	2	

Table 4 shows a summary of the regression models with EQ-5D-derived utility score as the dependent variable. An insignificant negative association between PGSI score and EQ-5D score was found both when comorbidities were controlled for and when they were not. (Model 1a and Model 1b). When compared to no-risk/non-gamblers, the PGSI categories had an insignificant negative association with EQ-5D utility score, both when comorbidities were controlled for and when they were not (Model 2a and Model 2b). A higher PGSI harm score was associated with a statistically lower EQ-5D score when no covariates were included in the model (Model 3a), with a lower utility of -0.008 associated with each 1-point increase of the derived harm score. When potential comorbidities were controlled for this association was no longer significant (Model 3b).

**Table 4:** Model summaries for the models where EQ-5D-derived utility score was the dependent variable

	Beta coefficients (SE)					
Dependent variable	EQ-5D-5L derived utility score					
Model number	Model 1a	Model 1b	Model 2a	Model 2b	Model 3a	Model 3b
PGSI score	-0.006 (0.003)	-0.003 (0.003)				
*PGSI categories (ref = no-risk/ non-gambler)*						
Low Risk			-0.018 (0.023)	-0.030 (0.020)		
Moderate Risk			-0.066 (0.035)	-0.052 (0.030)		
High Risk			-0.092 (0.050)	-0.051 (0.043)		
7-item PGSI derived harm variable					-0.008^*^ (0.004)	-0.005 (0.003)
Long-term mental disorder (ref = no)		-0.234^**^ (0.029)		-0.237^**^ (0.029)		-0.234^**^ (0.029)
Disability Allowance (ref = no)		-0.232^**^ (0.030)		-0.232^**^ (0.030)		-0.232^**^ (0.030)
*Frequency of alcohol intake (ref = non-drinker)*						
Almost every day		-0.002 (0.042)		0.008 (0.042)		-0.003 (0.042)
Five or six days a week		-0.011 (0.048)		0.001 (0.048)		-0.012 (0.048)
Three or four days a week		0.038 (0.038)		0.047 (0.039)		0.037 (0.038)
Once or twice a week		-0.024 (0.033)		-0.013 (0.033)		-0.025 (0.033)
Once or twice a month		0.010 (0.036)		0.020 (0.036)		0.009 (0.036)
Once every couple of months		0.017 (0.044)		0.028 (0.044)		0.016 (0.044)
Once or Twice a Year		0.056 (0.044)		0.063 (0.044)		0.055 (0.044)
*Cigarettes per day (ref* = *non-smoker)*						
> = 10		-0.049 (0.029)		-0.047 (0.029)		-0.048 (0.029)
< 10		-0.038 (0.028)		-0.039 (0.028)		-0.037 (0.028)
*Age (ref* = *16–19 years old)*						
20–24		-0.002 (0.050)		-0.004 (0.051)		-0.002 (0.050)
25–29		-0.016 (0.052)		-0.020 (0.052)		-0.016 (0.052)
30–34		-0.007 (0.048)		-0.011 (0.048)		-0.006 (0.048)
35–39		-0.010 (0.049)		-0.013 (0.049)		-0.008 (0.049)
40–44		-0.032 (0.051)		-0.033 (0.051)		-0.030 (0.051)
45–49		-0.113^*^ (0.051)		-0.116^*^ (0.051)		-0.112^*^ (0.051)
50–54		-0.079 (0.054)		-0.080 (0.055)		-0.077 (0.055)
55–59		-0.088 (0.052)		-0.092 (0.052)		-0.086 (0.052)
60–64		-0.118^*^ (0.060)		-0.120^*^ (0.060)		-0.116 (0.060)
65–69		-0.016 (0.075)		-0.021 (0.075)		-0.014 (0.075)
70–74		0.027 (0.066)		0.025 (0.066)		0.029 (0.066)
75–79		-0.203^*^ (0.080)		-0.205^*^ (0.080)		-0.202^*^ (0.080)
80–84		-0.256 (0.138)		-0.259 (0.137)		-0.253 (0.138)
85 +		-0.133 (0.101)		-0.130 (0.101)		-0.129 (0.101)
Male (ref = female)		0.023 (0.021)		0.024 (0.021)		0.023 (0.021)
*Ethnicity (ref* = *white)*						
Black		0.034 (0.056)		0.035 (0.056)		0.034 (0.056)
Asian		0.005 (0.042)		0.005 (0.042)		0.005 (0.042)
Mixed		0.003 (0.061)		0.015 (0.061)		0.002 (0.061)
Other		-0.068 (0.111)		-0.069 (0.111)		-0.068 (0.111)
Constant	0.825^**^ (0.012)	0.903^**^ (0.051)	0.833^**^ (0.015)	0.909^**^ (0.051)	0.825^**^ (0.011)	0.902^**^ (0.051)
Observations	472	442	472	442	472	442
R^2^	0.008	0.371	0.013	0.376	0.011	0.371
Adjusted R^2^	0.006	0.323	0.007	0.325	0.009	0.324
Residual Std. Error	0.228 (df = 470)	0.184 (df = 410)	0.228 (df = 468)	0.183 (df = 408)	0.228 (df = 470)	0.183 (df = 410)
F Statistic	3.636 (df = 1; 470)	7.798^**^ (df = 31; 410)	2.029 (df = 3; 468)	7.446^**^ (df = 33; 408)	5.332^*^ (df = 1; 470)	7.816^**^ (df = 31; 410)

^*^p < 0.05

**p < 0.001

Table 5 shows the results of the models with each EQ-5D domain as the dependent variable. PGSI score did not have a significant association with any of the EQ-5D domains (Model 4a-4e). The moderate risk PGSI category had a significant positive association with the pain domain of the EQ-5D, an increase of 0.325 in the pain domain score compared to no-risk/non-gamblers (Model 5d). A 1-point increase in the 7-item PGSI derived harms variable was associated with a statistically significant increase of 0.03 in the anxiety/depression domain score (Model 6e). Table 5.1 in Additional File 3 shows the full results of the EQ-5D domain analysis, including all the coefficients for the covariates included.

**Table 5:** Model summaries for the models where EQ-5D domain scores were the dependent variables

Beta coefficients (SE)															
Dependent variable	EQ-5D-5L Domain scores														
EQ-5D-5L Domain	M	SC	UA	P	AD	M	SC	UA	P	AD	M	SC	UA	P	AD
Model Number	4a	4b	4c	4d	4e	5a	5b	5c	5d	5e	6a	6b	6c	6d	6e
PGSI score	0.012 (0.011)	-0.005 (0.006)	-0.009 (0.009)	0.006 (0.013)	0.023 (0.012)										
*PGSI categories (ref = no-risk/ non-gambler)*															
Low Risk						0.073 (0.077)	-0.034 (0.042)	0.120 (0.068)	0.130 (0.092)	0.132 (0.087)					
Moderate Risk						0.130 (0.116)	0.005 (0.063)	0.050 (0.102)	0.325^*^ (0.139)	0.165 (0.131)					
High Risk						0.212 (0.166)	-0.060 (0.090)	-0.049 (0.146)	0.081 (0.199)	0.323 (0.187)					
7-item PGSI derived harm variable											0.014 (0.013)	-0.007 (0.007)	-0.012 (0.011)	0.010 (0.016)	0.030^*^ (0.015)
Other covariates included^a^	✓	✓	✓	✓	✓	✓	✓	✓	✓	✓	✓	✓	✓	✓	✓
Constant	1.230^**^ (0.194)	0.896^**^ (0.105)	1.358^**^ (0.171)	1.494^**^ (0.233)	1.228^**^ (0.218)	1.217^**^ (0.197)	0.909^**^ (0.107)	1.310^**^ (0.173)	1.480^**^ (0.235)	1.197^**^ (0.221)	1.235^**^ (0.194)	0.894^**^ (0.105)	1.355^**^ (0.171)	1.496^**^ (0.233)	1.236^**^ (0.218)
Observations	442	442	442	442	442	442	442	442	442	442	442	442	442	442	442
R^2^	0.271	0.307	0.370	0.217	0.347	0.273	0.307	0.374	0.228	0.349	0.271	0.307	0.370	0.218	0.348
Adjusted R^2^	0.216	0.255	0.322	0.158	0.298	0.215	0.251	0.323	0.166	0.297	0.216	0.255	0.322	0.159	0.299
Residual Std. Error	0.701 (df = 410)	0.380 (df = 410)	0.617 (df = 410)	0.843 (df = 410)	0.787 (df = 410)	0.701 (df = 408)	0.381 (df = 408)	0.617 (df = 408)	0.839 (df = 408)	0.788 (df = 408)	0.701 (df = 410)	0.380 (df = 410)	0.617 (df = 410)	0.843 (df = 410)	0.787 (df = 410)
F Statistic	4.917^**^ (df = 31; 410)	5.857^**^ (df = 31; 410)	7.752^**^ (df = 31; 410)	3.673^**^ (df = 31; 410)	7.030^**^ (df = 31; 410)	4.654^**^ (df = 33; 408)	5.485^**^ (df = 33; 408)	7.380^**^ (df = 33; 408)	3.659^**^ (df = 33; 408)	6.634^**^ (df = 33; 408)	4.910^**^ (df = 31; 410)	5.868^**^ (df = 31; 410)	7.762^**^ (df = 31; 410)	3.682^**^ (df = 31; 410)	7.062^**^ (df = 31; 410)

*Abbreviation*: *M* mobility, *SC* self-care, *UA* usual activities, *P* pain, *AD* anxiety/depression

^*^p < 0.05

^**^p < 0.001

^a^The other covariates include: Presence of long−term mental health disorder, disability allowance, frequency of alcohol intake, cigarettes smoked per day, age, sex and ethnicity. See Table 5.1 in Additional File 3 for a table displaying all the coefficients for these covariates

## Discussion

This is the first study in the UK to estimate EQ-5D-5L-derived utility values associated with varying levels of problem gambling severity and gambling-related harm. The models that did not control for comorbidities found a statistically significant relationship between EQ-5D-5L-derived utility score and the derived 7-item PGSI derived harm variable. However, when controlling for comorbidities this relationship was no longer statistically significant. The analysis of the EQ-5D-5L domain scores shows that an increase in the 7-item PGSI derived harm variable is significantly associated with an increase in the anxiety and depression domain scores. A significant association was also found between being in the moderate risk PGSI category and a higher score in the pain domain of the EQ-5D-5L.

### Strengths and limitations

A major strength of this work is the use of the HSE data from 2018. This is a large dataset which is representative of the English population, and one of the only datasets which collected data on both gambling behaviour and risk and HRQoL measured by EQ-5D [26]. This allowed us to repeat existing work from other countries within the UK setting for the first time.

There are several limitations of this analysis. This study had a small sample size, with 442 people included in the analytic sample for models which controlled for comorbidities. This was due to only one wave of the HSE (2018 wave) including both the EQ-5D and PGSI questions [26]. Furthermore, since this dataset is representative of the English population the PGSI data was highly skewed with a small percentage having a PGSI score of 1 + which reduced the sample size even further after propensity score matching was used. Previous studies which did find a significant association between problem gambling severity and HRQoL had much larger sample sizes. For example, Browne et al. 2022 and Moayeri 2020 had sample sizes of 2,603 and 15,144 respectively [14, 15]. This could explain why they found more consistently significant results in their analysis.

Whilst efforts were made to follow the framework developed by Browne et al. this could not be exactly done due to using secondary data [25]. For example, it was recommended that personality disorders be included as a covariate in the modelling yet the HSE did not have any data on this. It is unclear what impact this might have had on the results. A previous study did control for the presence of a personality disorder when looking at the relationship between gambling problems and HRQoL [15]. They did not find a significant relationship between personality disorder and HRQoL when controlling for other potential comorbidities.

A limitation, which is not specific to this analysis, is the use of the PGSI as a proxy for gambling-related harm. The PGSI score measures both gambling behaviour and some, but crucially not all, of the negative consequences of gambling [16]. The analysis considered this issue by creating a new variable which was a summary measure of the PGSI items related to the negative consequences. The 7-item derived harm variable did have a significant association with EQ-5D score which could indicate it was better at measuring the negative consequences of gambling than the full PGSI score. In 2018, Browne et al. developed the Short Gambling Harm Screen (SGHS), a screening tool designed to screen for the presence and severity of gambling-related harms [31]. Browne et al. found that the PGSI and the SGHS both estimated similar levels of gambling-related harm, conceptualised as HRQoL, measured using the SF-6D [15]. Consequently, it is unlikely that the insignificant results in this analysis are due to problems with the PGSI not detecting levels of gambling-related harm.

The previous studies which found a statistically significant relationship between problem gambling severity (defined using the PGSI) and HRQoL used the SF-6D rather than the EQ-5D to measure HRQoL [14, 15, 32]. It could be that the EQ-5D is an inappropriate instrument to measure the change in HRQoL due to gambling. The only significant results in the analysis of the EQ-5D domains were between the anxiety/depression domain and the 7-item derived harms variable, and the pain domain with the moderate-risk PGSI category. It was expected that the anxiety/depression domain of the EQ-5D would be most significantly associated with problem gambling severity, since poor mental health is a well-recognised consequence of problems with gambling [21]. The significant relationship between the moderate risk PGSI category and the pain domain of the EQ-5D was unexpected and is likely a result of the small sample size in this study. Alternative HRQoL instruments which have domains measuring other areas of HRQoL might be more appropriate to use in gambling studies. For example, unlike the SF-6D, the EQ-5D does not assess the impact on social relationships [11]. Relationship disruption, conflict or breakdown were identified as harms in Langham’s taxonomy of gambling-related harms [21]. This could mean that a measure, such as the SF-6D which does assess more of the potential gambling harms, could be more appropriate than the EQ-5D. The EuroQol group which developed the EQ-5D are currently developing a similar self-reported outcome measure, the EQ Health and Wellbeing instrument (EQ-HWB), which takes a broader approach and will be suitable to be used across both health and social care sectors [33]. The EQ-HWB has additional domains to the EQ-5D, including domains such as energy (feeling tired), cognition (trouble concentrating) and a social relationships domain (feeling lonely). This measure is still in the experimental phase but has the potential to be used to measure the impact of gambling on HRQoL.

There are broader measures that have been used to measure quality of life, rather than the HRQoL, in gambling studies [22]. These include the World Health Organization Quality of Life Assessment Instrument, The Quality-of-Life Enjoyment and Satisfaction Questionnaire, The Quality of Life Inventory and The Personal Well-Being Index [34–37]. All of these instruments, except The Personal Well-Being Index, assess financial concerns. Much gambling-related harm occurs due to financial harms and so it is likely that measures which assess financial concerns will be more sensitive to decreases in quality of life due to changes in gambling severity [21, 38]. Depending on the perspective of the decision maker for policies/interventions it could be more appropriate to look at these broader measures of quality of life rather than HRQoL. For example, governments might be more interested in broader measures of quality of life whereas healthcare providers will be more concerned with HRQoL. Future research could look at these measures, and the SF-6D and EQ-WHB discussed above to see how they can be used to quantify gambling-related harm.

When using indirect methods to elicit health state utilities, it is essential to control for comorbidities in the statistical models to try and isolate the impact of gambling on HRQoL [25]. Mental health disorders such as anxiety and depression can result from gambling and therefore by controlling for these the impact of gambling on HRQoL would be underestimated [21]. Interaction terms could be used in regression models to understand the impact of including variables for comorbidities as suggested in the Browne et al. framework [25]. For example, an interaction term between PGSI score and mental health disorder would inform us about how the presence of mental health disorder impacts the PGSI score. The small sample size in this study meant it was not possible to include interaction terms. The impact of interaction terms could be investigated using larger samples, such as the HILDA sample used by Moayeri in 2019 and the primary data collected by Browne et al. in their 2022 study [14, 15].

## Conclusion

When controlling for comorbidities this study found no associations between problem gambling severity and HRQoL. This could reflect four possibilities. Firstly, this finding might reflect reality and there is no significant association between problem gambling severity and HRQoL, however the results of previous studies dispute this. There might be a significant association, but our relatively small sample size did not allow us to detect this. The PGSI might not be able to capture the harm caused by gambling, although previous studies have found significant associations between PGSI score and HRQoL. The final possibility is that the EQ-5D is not sensitive enough to detect the changes in HRQoL due to gambling. Future research, involving larger sample sizes than used in this analysis, should explore these possibilities so the relationship between problem gambling severity and HRQoL can be further understood.

### Supplementary Information

**Additional file 1.**

** Additional file 2.**

** Additional file 3.**

## Data Availability

The 2018 Health Survey for England dataset is available from the UK Data Service: NatCen Social Research, University College London, Department of Epidemiology and Public Health. (2022). *Health Survey for England, 2018*. [data collection]. *2nd Edition.* UK Data Service. SN: 8649, DOI: http://doi.org/10.5255/UKDA-SN-8649-2. The code to run this analysis is available at https://github.com/esther-moore/PGSI_HRQoL. The Microsoft Excel macro used to calculate the EQ-5D scores is available from the NICE Decision Support Unit at https://www.sheffield.ac.uk/nice-dsu/methods-development/mapping-eq-5d-5l-3l.

## Abbreviations

AIC: Akaike Information Criteria
CSE: Certificate of Secondary Education
EQ-5D: EuroQol-5D
EQ-5D-5L: EuroQol-5D 5 level version
EQ-HWB: EuroQol-Health and Wellbeing instrument
GCE: General Certificate of Education
HILDA: Household, Income and Labour Dynamics in Australia (HILDA) Survey
HRQoL: Health-related Quality of Life
HSE: Health Survey for England
NHS: National Health Service
NICE: National Institute for Health and Care Excellence
NS-SEC: National Statistics Socio-economic classification
NVQ: National Vocational Qualification
OLS: Ordinary Least Squares
PGSI: Problem Gambling Severity Index
QALY: Quality-Adjusted Life Years
*SE*: Standard Error
SF-36: Short Form-36
SF-6D: Short-Form Six-Dimension
SGHS: Short Gambling Harm Screen

## References

[1] Johnstone P, Regan M (2020). Gambling harm is everybody’s business: a public health approach and call to action. Public Health.

[2] Health TLP (2021). Gambling: a neglected public health issue. Lancet Public Health.

[3] Office For Health Improvement and Disparities (2023). The economic cost of gambling-related harm in England: evidence update 2023.

[4] National Institute for Health and Care Excellence. Project information | Harmful gambling: identification, assessment and management | Guidance | NICE. NICE; 2024. Available from: https://www.nice.org.uk/guidance/indevelopment/gid-ng10210. Cited 2023 Apr 27.

[5] Policies and interventions to reduce harmful gambling: an international Delphi consensus and implementation rating study | Elsevier Enhanced Reader. Available from: https://reader.elsevier.com/reader/sd/pii/S2468266722001372?token=A3D9962E0D59CD0F9068B83D7E1A9E944BAF849096264AA9FF26098C0CCFBD661CE9E9076E289E42B943057D48B6B42E&originRegion=eu-west-1&originCreation=20220728151339. Cited 2022 Jul 28. 10.1016/S2468-2667(22)00137-2 35907421

[6] The NHS Long Term Plan. 2019. Available from: https://www.longtermplan.nhs.uk/. Cited 2023 Sep 6.

[7] NHS England » NHS launches new gambling addiction clinics to meet record demand. Available from: https://www.england.nhs.uk/2022/02/nhs-launches-new-gambling-addiction-clinics-to-meet-record-demand/. Cited 2023 Jan 19.

[8] 7 Assessing cost effectiveness | The guidelines manual | Guidance | NICE. NICE; 2012. Available from: https://www.nice.org.uk/process/pmg6/chapter/assessing-cost-effectiveness. Cited 2023 Sep 5.

[9] Neumann P. Cost-effectiveness in health and medicine. Second edition. New York, NY, United States of America: Oxford University Press; 2017. 10.1093/acprof:oso/9780190492939.001.0001. Cited 2023 Sep 5.

[10] Arnold D, Girling A, Stevens A, Lilford R (2009). Comparison of direct and indirect methods of estimating health state utilities for resource allocation: review and empirical analysis. BMJ.

[11] Brazier J, Roberts J, Tsuchiya A, Busschbach J (2004). A comparison of the EQ-5D and SF-6D across seven patient groups. Health Econ.

[12] Browne M, Bellringer M, Greer N, Kolandai-Matchett K, Rawat V, Langham E, Rockloff M, et al. Measuring the burden of gambling harm in New Zealand. Wellington: Ministry of Health; 2017.

[13] Rawat V, Browne M, Bellringer M, Greer N, Kolandai-Matchett K, Rockloff M (2018). A tale of two countries: comparing disability weights for gambling problems in New Zealand and Australia. Qual Life Res.

[14] Moayeri F (2020). A reference set of Health State Utility Values for gambling problem behaviour, a survey of the Australian general population: implications for future healthcare evaluations. Expert Rev Pharmacoecon Outcomes Res.

[15] Matthew Browne, Alex M. T. Russell, Stephen Begg, Matthew J. Rockloff, Vijay Rawat, Nerilee Hing, et al. Benchmarking gambling screens to health-state utility: the PGSI and the SGHS estimate similar levels of population gambling-harm | SpringerLink. 2022. Available from: https://link.springer.com/article/10.1186/s12889-022-13243-4. Cited 2022 Jun 28. 10.1186/s12889-022-13243-4 PMC9044680 35473621

[16] Wynne HJ (2002). Introducing the Canadian problem gambling index.

[17] Harris S, Pockett RD, Dighton G, Wood K, Armour C, Fossey M, et al. Social and economic costs of gambling problems and related harm among UK military veterans. BMJ Mil Health. 2021. Available from: https://militaryhealth.bmj.com/content/early/2022/04/03/bmjmilitary-2021-001892. Cited 2023 Jan 19. 10.1136/bmjmilitary-2021-001892 34663678

[18] NICE [Internet]. NICE; [cited 2023 Jan 25]. Health technology evaluation manual | Technology appraisal guidance | NICE guidance | Our programmes | What we do | About. Available from: https://www.nice.org.uk/about/what-we-do/our-programmes/nice-guidance/nice-technology-appraisal-guidance/changes-to-health-technology-evaluation.

[19] Herdman M, Gudex C, Lloyd A, Janssen M, Kind P, Parkin D (2011). Development and preliminary testing of the new five-level version of EQ-5D (EQ-5D-5L). Qual Life Res Int J Qual Life Asp Treat Care Rehabil.

[20] Devlin NJ, Shah KK, Feng Y, Mulhern B, van Hout B (2018). Valuing health-related quality of life: An EQ-5D-5L value set for England. Health Econ.

[21] Langham E, Thorne H, Browne M, Donaldson P, Rose J, Rockloff M (2015). Understanding gambling related harm: a proposed definition, conceptual framework, and taxonomy of harms. BMC Public Health.

[22] Bonfils NA, Aubin HJ, Benyamina A, Limosin F, Luquiens A (2019). Quality of life instruments used in problem gambling studies: a systematic review and a meta-analysis. Neurosci Biobehav Rev.

[23] Subramaniam M, Abdin E, Vaingankar JA, Wong KE, Chong SA (2015). Comorbid physical and mental illnesses among pathological gamblers: results from a population based study in Singapore. Psychiatry Res.

[24] Lahti T, Halme JT, Pankakoski M, Sinclair D, Alho H (2010). Treatment of pathological gambling with naltrexone pharmacotherapy and brief intervention: a pilot study. Psychopharmacol Bull.

[25] Browne M, Rawat V, Newall P, Begg S, Rockloff M, Hing N (2020). A framework for indirect elicitation of the public health impact of gambling problems. BMC Public Health.

[26] NatCen Social Research, UCL. Health Survey for England 2018 Methods. NHS digital. 2019. Available from: https://files.digital.nhs.uk/CA/2393EF/HSE18-Methods-rep.pdf.

[27] Raisamo SU, Mäkelä P, Salonen AH, Lintonen TP (2015). The extent and distribution of gambling harm in Finland as assessed by the problem gambling severity index. Eur J Public Health.

[28] Young MM, Hodgins DC, Currie SR, Brunelle N, Dufour M, Flores-Pajot MC, et al. Not too much, not too often, and not too many: the results of the first large-scale, international project to develop lower-risk gambling guidelines. Int J Ment Health Addict. 2022. 10.1007/s11469-022-00896-w. Cited 2022 Aug 22.

[29] Position statement on use of the EQ-5D-5L value set for England (updated October 2019) | Technology appraisal guidance | NICE guidance | Our programmes | What we do | About. NICE. NICE. Available from: https://www.nice.org.uk/about/what-we-do/our-programmes/nice-guidance/technology-appraisal-guidance/eq-5d-5l. Cited 2023 Jan 20.

[30] Hernández Alava M, Pudney S, Wailoo A. Estimating the Relationship Between EQ-5D-5L and EQ-5D-3L: Results from a UK Population Study. PharmacoEconomics. 2022. Available from: 10.1007/s40273-022-01218-7. Cited 2023 Jan 20. 10.1007/s40273-022-01218-7 PMC9883358 36449173

[31] Browne M, Goodwin BC, Rockloff MJ (2018). Validation of the Short Gambling Harm Screen (SGHS): a tool for assessment of harms from gambling. J Gambl Stud.

[32] Tulloch C, Hing N, Browne M, Rockloff M (2023). How gambling problems relate to health and wellbeing in Australian households: evidence from the household income and labour dynamics of Australia survey. Addict Behav.

[33] Brazier J, Peasgood T, Mukuria C, Marten O, Kreimeier S, Luo N (2022). The EQ-HWB: overview of the development of a measure of health and wellbeing and key results. Value Health J Int Soc Pharmacoeconomics Outcomes Res.

[34] WHOQOL - Measuring Quality of Life| The World Health Organization [Internet]. [cited 2023 Oct 10]. Available from: https://www.who.int/tools/whoqol.

[35] J E, J N, W H, R B. Quality of Life Enjoyment and Satisfaction Questionnaire: a new measure. Psychopharmacol Bull. 1993;29(2):321–6. 8290681

[36] Frisch MB, Cornell J, Villanueva M, Retzlaff PJ. Clinical validation of the Quality of Life Inventory. A measure of life satisfaction for use in treatment planning and outcome assessment. Psychol Assess. 1992;4(1):92–101.

[37] Cummins RA, Eckersley R, Pallant J, Van Vugt J, Misajon R. Developing a national index of subjective wellbeing: The Australian Unity Wellbeing Index. Soc Indic Res. 2003;64(2):159–90.

[38] Swanton TB, Gainsbury SM (2020). Debt stress partly explains the relationship between problem gambling and comorbid mental health problems. Soc Sci Med.

