# Supplementary material for: The association between health-related quality of life and problem gambling severity: a cross-sectional analysis of the Health Survey for England
Source: BMC Public Health. 2024 Feb 12;24:434. doi: 10.1186/s12889-024-17816-3 (PMC10860212; doi:10.1186/s12889-024-17816-3)
Supplement: Supplementary file 1 — Additional file 1. [file 12889_2024_17816_MOESM1_ESM.docx]

**Problem Gambling Severity Index** **(PGSI)**

Responses to each item are given the following scores:

- never = zero
- sometimes = one
- most of the time = two
- almost always = three

Thinking about the last 12 months…

1. Have you bet more than you could really afford to lose?

0 Never 1 Sometimes 2 Most of the time 3 Almost always

1. Have you needed to gamble with larger amounts of money to get the same feeling of excitement?

0 Never 1 Sometimes 2 Most of the time 3 Almost always

1. When you gambled, did you go back another day to try to win back the money you lost?

0 Never 1 Sometimes 2 Most of the time 3 Almost always

1. Have you borrowed money or sold anything to get money to gamble?

0 Never 1 Sometimes 2 Most of the time 3 Almost always

1. Have you felt that you might have a problem with gambling?

0 Never 1 Sometimes 2 Most of the time 3 Almost always

1. Has gambling caused you any health problems, including stress or anxiety?

0 Never 1 Sometimes 2 Most of the time 3 Almost always

1. Have people criticized your betting or told you that you had a gambling problem, regardless of whether or not you thought it was true?

0 Never 1 Sometimes 2 Most of the time 3 Almost always

1. Has your gambling caused any financial problems for you or your household?

0 Never 1 Sometimes 2 Most of the time 3 Almost always

1. Have you felt guilty about the way you gamble or what happens when you gamble?

0 Never 1 Sometimes 2 Most of the time 3 Almost always

Scoring instructions:

0 No problem gamblers

1-2 Low risk gamblers

3-7 Moderate risk gamblers

8 or more High risk gamblers

Ferris, J., & Wynne, H. (2001). The Canadian problem gambling index: Final report. Submitted for the Canadian Centre on Substance Abuse.
