# Supplementary material for: The association between health-related quality of life and problem gambling severity: a cross-sectional analysis of the Health Survey for England
Source: BMC Public Health. 2024 Feb 12;24:434. doi: 10.1186/s12889-024-17816-3 (PMC10860212; doi:10.1186/s12889-024-17816-3)
Supplement: Supplementary file 2 — Additional file 2. [file 12889_2024_17816_MOESM2_ESM.docx]

Table 1.1 Demographic characteristics of the sample after propensity score matching

|  | **PGSI 0 N(%)** | **PGSI 1+ N(%)** | ***P* Value** |
| --- | --- | --- | --- |
| N | 236 (50%) | 236 (50%) |  |
| Sex |  |  | >0.999 |
| Male | 175 (74.15%) | 175 (74.15%) |  |
| Female | 61 (25.85%) | 61 (25.85%) |  |
| Age (years) |  |  | >0.999 |
| 16-29 | 60 (25.42%) | 60 (25.42%) |  |
| 30-44 | 90 (38.14%) | 90 (38.14%) |  |
| 45-59 | 57 (24.15%) | 57 (24.15%) |  |
| 60-74 | 22 (9.32%) | 22 (9.32%) |  |
| 75+ | 7 (2.97%) | 7 (2.97%) |  |
| Ethnicity |  |  | 0.044 |
| White | 203 (86.02%) | 211 (89.41%) |  |
| Black | 9 (3.81%) | 6 (2.54%) |  |
| Asian | 19 (8.05%) | 9 (3.81%) |  |
| Mixed | 2 (0.85%) | 9 (3.81%) |  |
| Other | 3 (1.27%) | 1 (0.42%) |  |
| Highest educational qualification |  |  | >0.999 |
| NVQ4/NVQ5/Degree or equiv | 47 (19.92%) | 47 (19.92%) |  |
| Higher ed below degree | 31 (13.14%) | 31 (13.14%) |  |
| NVQ3/GCE A Level equiv | 61 (25.85%) | 61 (25.85%) |  |
| NVQ2/GCE O Level equiv | 48 (20.34%) | 48 (20.34%) |  |
| NVQ1/CSE other grade equiv | 8 (3.39%) | 8 (3.39%) |  |
| Foreign/other | 1 (0.42%) | 1 (0.42%) |  |
| No qualification | 40 (16.95%) | 40 (16.95%) |  |
| NS-SEC (occupation) |  |  | 0.202 |
| Higher managerial and professional occupations | 23 (9.96%) | 21 (8.94%) |  |
| Lower managerial and professional occupations | 45 (19.48%) | 50 (21.28%) |  |
| Intermediate occupations | 24 (10.39%) | 34 (14.47%) |  |
| Small employers and own account workers | 36 (15.58%) | 25 (10.64%) |  |
| Lower supervisory and technical occupations | 27 (11.69%) | 18 (7.66%) |  |
| Semi-routine occupations | 38 (16.45%) | 39 (16.60%) |  |
| Routine occupations | 24 (10.39%) | 38 (16.17%) |  |
| Never worked and long-term unemployed | 6 (2.60%) | 2 (0.85%) |  |
| Other | 8 (3.46%) | 8 (3.40%) |  |
| Missing | 5 | 1 |  |
| Marital status |  |  | 0.635 |
| Single | 63 (26.69%) | 79 (33.47%) |  |
| Married, including civil partnership | 92 (38.98%) | 83 (35.17%) |  |
| Separated, including from civil partnership | 4 (1.69%) | 4 (1.69%) |  |
| Divorced, including dissolved civil partnership | 13 (5.51%) | 16 (6.78%) |  |
| Widowed, including civil partnership | 7 (2.97%) | 6 (2.54%) |  |
| Cohabitees | 57 (24.15%) | 48 (20.34%) |  |
| Income (Equivalised Income Quintiles) |  |  | 0.805 |
| Highest Quintile (>£52,817) | 40 (21.62%) | 46 (22.55%) |  |
| Second highest Quintile (>£31,967 <=£52,817) | 29 (15.68%) | 32 (15.69%) |  |
| Middle Quintile (>£23,084 <=£31,967) | 23 (12.43%) | 31 (15.20%) |  |
| Second lowest Quintile (>£14,918 <= £23,084) | 51 (27.57%) | 46 (22.55%) |  |
| Lowest Quintile (<=£14,918) | 42 (22.70%) | 49 (24.02%) |  |
| Missing | 51 | 32 |  |
| Residence |  |  | 0.004 |
| Rural | 56 (23.73%) | 31 (13.14%) |  |
| Urban | 180 (76.27%) | 205 (86.86%) |  |
| Long lasting condition affecting behaviour and social skills |  |  | 0.57 |
| Yes | 14 (14.89%) | 11 (11.11%) |  |
| No | 80 (85.11%) | 88 (88.89%) |  |
| Missing | 3598 | 137 |  |
