# Supplementary material for: The association between health-related quality of life and problem gambling severity: a cross-sectional analysis of the Health Survey for England
Source: BMC Public Health. 2024 Feb 12;24:434. doi: 10.1186/s12889-024-17816-3 (PMC10860212; doi:10.1186/s12889-024-17816-3)
Supplement: Supplementary file 3 — Additional file 3. [file 12889_2024_17816_MOESM3_ESM.docx]

Table 5.1 Model summaries for the models where EQ-5D domain scores were the dependent variables, displaying the coefficients for all included variables.

| Beta coefficients (SE) | | | | | | | | | | | | | | | |
| --- | --- | --- | --- | --- | --- | --- | --- | --- | --- | --- | --- | --- | --- | --- | --- |
| Dependent variable | EQ-5D-5L Domain scores | | | | | | | | | | | | | | |
| EQ-5D-5L Domain | M | SC | UA | P | AD | M | SC | UA | P | AD | M | SC | UA | P | AD |
| Model Number | 4a | 4b | 4c | 4d | 4e | 5a | 5b | 5c | 5d | 5e | 6a | 6b | 6c | 6d | 6e |
| PGSI score | 0.012 (0.011) | -0.005 (0.006) | -0.009 (0.009) | 0.006 (0.013) | 0.023 (0.012) |  |  |  |  |  |  |  |  |  |  |
| *PGSI categories*  *(ref= no-risk/ non-gambler)* | | | | | | | | | | | | | | | |
| Low Risk |  |  |  |  |  | 0.073 (0.077) | -0.034 (0.042) | 0.120 (0.068) | 0.130 (0.092) | 0.132 (0.087) |  |  |  |  |  |
| Moderate Risk |  |  |  |  |  | 0.130 (0.116) | 0.005 (0.063) | 0.050 (0.102) | 0.325^*^ (0.139) | 0.165 (0.131) |  |  |  |  |  |
| High Risk |  |  |  |  |  | 0.212 (0.166) | -0.060 (0.090) | -0.049 (0.146) | 0.081 (0.199) | 0.323 (0.187) |  |  |  |  |  |
| 7-item PGSI derived harm variable |  |  |  |  |  |  |  |  |  |  | 0.014 (0.013) | -0.007 (0.007) | -0.012 (0.011) | 0.010 (0.016) | 0.030^*^ (0.015) |
| Long-term mental disorder (ref=no) | 0.242^*^ (0.110) | 0.150^*^ (0.059) | 0.576^**^ (0.097) | 0.438^**^ (0.132) | 1.431^**^ (0.123) | 0.246^*^ (0.110) | 0.151^*^ (0.060) | 0.580^**^ (0.097) | 0.462^**^ (0.132) | 1.436^**^ (0.124) | 0.242^*^ (0.110) | 0.150^*^ (0.059) | 0.576^**^ (0.096) | 0.437^**^ (0.132) | 1.430^**^ (0.123) |
| Disability Allowance (ref=no) | 0.819^**^ (0.116) | 0.609^**^ (0.063) | 0.758^**^ (0.102) | 0.621^**^ (0.139) | 0.487^**^ (0.130) | 0.819^**^ (0.116) | 0.605^**^ (0.063) | 0.773^**^ (0.102) | 0.612^**^ (0.139) | 0.490^**^ (0.131) | 0.817^**^ (0.116) | 0.609^**^ (0.063) | 0.758^**^ (0.102) | 0.623^**^ (0.139) | 0.487^**^ (0.130) |
| *Frequency of alcohol intake*  *(ref=non-drinker)* | | | | | | | | | | | | | | | |
| Almost every day | -0.098 (0.160) | 0.047 (0.087) | -0.322^*^ (0.141) | 0.261 (0.193) | -0.074 (0.180) | -0.120 (0.162) | 0.047 (0.088) | -0.346^*^ (0.142) | 0.198 (0.194) | -0.102 (0.182) | -0.093 (0.160) | 0.045 (0.087) | -0.325^*^ (0.141) | 0.263 (0.193) | -0.066 (0.180) |
| Five or six days a week | -0.109 (0.182) | 0.061 (0.099) | -0.264 (0.160) | 0.276 (0.219) | -0.069 (0.204) | -0.136 (0.184) | 0.066 (0.100) | -0.308 (0.162) | 0.209 (0.220) | -0.109 (0.207) | -0.105 (0.182) | 0.060 (0.098) | -0.267 (0.160) | 0.277 (0.218) | -0.062 (0.204) |
| Three or four days a week | -0.241 (0.146) | 0.020 (0.079) | -0.334^*^ (0.128) | 0.060 (0.175) | -0.150 (0.164) | -0.259 (0.148) | 0.027 (0.080) | -0.372^*^ (0.130) | 0.008 (0.177) | -0.183 (0.166) | -0.237 (0.146) | 0.018 (0.079) | -0.338^*^ (0.128) | 0.063 (0.175) | -0.142 (0.164) |
| Once or twice a week | -0.075 (0.125) | 0.073 (0.068) | -0.221^*^ (0.110) | 0.138 (0.151) | 0.093 (0.141) | -0.098 (0.128) | 0.074 (0.069) | -0.264^*^ (0.112) | 0.068 (0.153) | 0.063 (0.144) | -0.071 (0.125) | 0.072 (0.068) | -0.223^*^ (0.110) | 0.138 (0.150) | 0.098 (0.140) |
| Once or twice a month | -0.180 (0.137) | 0.040 (0.074) | -0.267^*^ (0.121) | 0.118 (0.165) | -0.066 (0.154) | -0.202 (0.139) | 0.042 (0.076) | -0.297^*^ (0.122) | 0.053 (0.167) | -0.096 (0.157) | -0.176 (0.137) | 0.039 (0.074) | -0.270^*^ (0.121) | 0.120 (0.165) | -0.058 (0.154) |
| Once every couple of months | -0.183 (0.168) | 0.110 (0.091) | -0.229 (0.148) | 0.105 (0.202) | -0.114 (0.189) | -0.206 (0.170) | 0.119 (0.092) | -0.271 (0.150) | 0.052 (0.204) | -0.155 (0.191) | -0.180 (0.168) | 0.109 (0.091) | -0.231 (0.148) | 0.107 (0.202) | -0.109 (0.189) |
| Once or Twice a Year | -0.273 (0.167) | -0.117 (0.090) | -0.402^*^ (0.147) | 0.113 (0.200) | -0.331 (0.187) | -0.287 (0.168) | -0.111 (0.091) | -0.427^*^ (0.147) | 0.072 (0.201) | -0.357 (0.188) | -0.272 (0.167) | -0.118 (0.090) | -0.404^*^ (0.147) | 0.115 (0.200) | -0.326 (0.187) |
| *Cigarettes per day (ref=non-smoker)* | | | | | | | | | | | | | | | |
| >= 10 per day | 0.213 (0.110) | 0.113 (0.059) | 0.246^*^ (0.097) | 0.217 (0.132) | 0.042 (0.123) | 0.210 (0.110) | 0.112 (0.060) | 0.221^*^ (0.096) | 0.200 (0.131) | 0.040 (0.123) | 0.215 (0.110) | 0.114 (0.059) | 0.247^*^ (0.097) | 0.214 (0.132) | 0.040 (0.123) |
| <10 per day | 0.101 (0.108) | 0.024 (0.058) | 0.268^*^ (0.095) | 0.125 (0.130) | 0.106 (0.121) | 0.105 (0.108) | 0.022 (0.059) | 0.263^*^ (0.095) | 0.129 (0.129) | 0.113 (0.121) | 0.101 (0.108) | 0.025 (0.059) | 0.270^*^ (0.095) | 0.122 (0.130) | 0.102 (0.121) |
| *Age (ref=16-19 years old)* | | | | | | | | | | | | | | | |
| 20-24 | -0.067 (0.192) | 0.098 (0.104) | -0.052 (0.169) | -0.225 (0.231) | 0.219 (0.216) | -0.066 (0.194) | 0.086 (0.105) | -0.023 (0.171) | -0.228 (0.232) | 0.235 (0.218) | -0.068 (0.192) | 0.099 (0.104) | -0.051 (0.169) | -0.227 (0.231) | 0.216 (0.216) |
| 25-29 | -0.178 (0.199) | 0.028 (0.108) | 0.035 (0.175) | -0.140 (0.239) | 0.165 (0.224) | -0.173 (0.200) | 0.019 (0.109) | 0.061 (0.176) | -0.131 (0.239) | 0.183 (0.225) | -0.180 (0.199) | 0.029 (0.108) | 0.037 (0.175) | -0.143 (0.239) | 0.160 (0.224) |
| 30-34 | -0.033 (0.182) | 0.081 (0.098) | -0.094 (0.160) | -0.160 (0.218) | 0.174 (0.204) | -0.027 (0.183) | 0.073 (0.099) | -0.066 (0.161) | -0.147 (0.219) | 0.190 (0.205) | -0.036 (0.182) | 0.083 (0.098) | -0.091 (0.160) | -0.163 (0.218) | 0.165 (0.204) |
| 35-39 | -0.098 (0.186) | 0.038 (0.101) | -0.093 (0.164) | -0.202 (0.224) | 0.247 (0.209) | -0.089 (0.187) | 0.027 (0.102) | -0.068 (0.165) | -0.196 (0.224) | 0.271 (0.211) | -0.103 (0.186) | 0.041 (0.101) | -0.089 (0.164) | -0.207 (0.224) | 0.236 (0.209) |
| 40-44 | -0.034 (0.193) | -0.0004 (0.105) | -0.039 (0.170) | -0.016 (0.232) | 0.329 (0.217) | -0.032 (0.195) | -0.010 (0.106) | -0.015 (0.171) | -0.027 (0.233) | 0.342 (0.219) | -0.039 (0.193) | 0.002 (0.105) | -0.034 (0.170) | -0.020 (0.232) | 0.317 (0.217) |
| 45-49 | 0.370 (0.193) | 0.184 (0.105) | 0.173 (0.170) | 0.458^*^ (0.232) | 0.160 (0.217) | 0.371 (0.195) | 0.176 (0.106) | 0.203 (0.171) | 0.465^*^ (0.233) | 0.172 (0.219) | 0.366 (0.193) | 0.187 (0.105) | 0.178 (0.170) | 0.453 (0.233) | 0.148 (0.217) |
| 50-54 | 0.157 (0.208) | 0.230^*^ (0.113) | 0.068 (0.183) | 0.170 (0.250) | 0.243 (0.234) | 0.158 (0.209) | 0.218 (0.114) | 0.088 (0.184) | 0.153 (0.251) | 0.257 (0.235) | 0.152 (0.208) | 0.234^*^ (0.113) | 0.073 (0.183) | 0.163 (0.250) | 0.229 (0.234) |
| 55-59 | 0.274 (0.200) | 0.089 (0.108) | 0.360^*^ (0.176) | 0.384 (0.240) | 0.124 (0.224) | 0.279 (0.201) | 0.078 (0.109) | 0.379^*^ (0.176) | 0.388 (0.240) | 0.145 (0.225) | 0.269 (0.200) | 0.093 (0.108) | 0.367^*^ (0.176) | 0.376 (0.241) | 0.108 (0.225) |
| 60-64 | 0.420 (0.227) | 0.219 (0.123) | 0.360 (0.200) | 0.235 (0.274) | 0.357 (0.256) | 0.426 (0.228) | 0.209 (0.124) | 0.381 (0.201) | 0.233 (0.273) | 0.377 (0.257) | 0.414 (0.228) | 0.222 (0.123) | 0.366 (0.200) | 0.230 (0.274) | 0.343 (0.256) |
| 65-69 | 0.102 (0.285) | 0.074 (0.154) | -0.002 (0.251) | 0.049 (0.343) | 0.114 (0.320) | 0.113 (0.285) | 0.070 (0.155) | 0.021 (0.251) | 0.072 (0.341) | 0.133 (0.321) | 0.095 (0.285) | 0.078 (0.154) | 0.003 (0.251) | 0.045 (0.342) | 0.100 (0.320) |
| 70-74 | 0.067 (0.250) | -0.108 (0.136) | -0.128 (0.220) | -0.058 (0.301) | -0.319 (0.281) | 0.071 (0.251) | -0.117 (0.136) | -0.112 (0.221) | -0.065 (0.300) | -0.302 (0.282) | 0.063 (0.251) | -0.105 (0.136) | -0.124 (0.220) | -0.063 (0.301) | -0.329 (0.281) |
| 75-79 | 0.821^*^ (0.305) | 0.142 (0.165) | 0.971^**^ (0.269) | 0.584 (0.367) | 0.224 (0.343) | 0.823^*^ (0.306) | 0.140 (0.166) | 0.985^**^ (0.269) | 0.597 (0.365) | 0.230 (0.343) | 0.817^*^ (0.305) | 0.144 (0.165) | 0.974^**^ (0.269) | 0.581 (0.367) | 0.215 (0.343) |
| 80-84 | 0.565 (0.526) | 0.775^*^ (0.285) | 0.639 (0.463) | 1.255^*^ (0.632) | -0.389 (0.591) | 0.571 (0.526) | 0.774^*^ (0.286) | 0.649 (0.462) | 1.281^*^ (0.629) | -0.380 (0.591) | 0.556 (0.526) | 0.779^*^ (0.285) | 0.647 (0.463) | 1.248^*^ (0.632) | -0.408 (0.590) |
| 85+ | 1.136^*^ (0.386) | 0.113 (0.209) | 0.911^*^ (0.340) | -0.018 (0.465) | 0.062 (0.434) | 1.130^*^ (0.388) | 0.099 (0.211) | 0.930^*^ (0.341) | -0.061 (0.464) | 0.070 (0.436) | 1.127^*^ (0.387) | 0.118 (0.210) | 0.919^*^ (0.340) | -0.026 (0.465) | 0.041 (0.434) |
| Male (ref=female) | -0.0004 (0.080) | -0.003 (0.043) | -0.071 (0.070) | -0.152 (0.096) | 0.014 (0.090) | -0.004 (0.080) | -0.004 (0.043) | -0.071 (0.070) | -0.162 (0.096) | 0.011 (0.090) | -0.001 (0.080) | -0.002 (0.043) | -0.071 (0.070) | -0.152 (0.096) | 0.014 (0.090) |
| *Ethnicity (ref=white)* | | | | | | | | | | | | | | | |
| Black | -0.066 (0.213) | -0.067 (0.115) | -0.115 (0.187) | 0.157 (0.256) | -0.163 (0.239) | -0.066 (0.213) | -0.069 (0.116) | -0.128 (0.187) | 0.149 (0.255) | -0.162 (0.239) | -0.065 (0.213) | -0.067 (0.115) | -0.115 (0.187) | 0.155 (0.256) | -0.164 (0.239) |
| Asian | -0.011 (0.161) | 0.116 (0.087) | -0.115 (0.142) | -0.064 (0.193) | -0.033 (0.181) | -0.010 (0.161) | 0.114 (0.088) | -0.105 (0.142) | -0.071 (0.193) | -0.032 (0.181) | -0.011 (0.161) | 0.115 (0.087) | -0.117 (0.142) | -0.061 (0.193) | -0.030 (0.181) |
| Mixed | 0.084 (0.232) | 0.126 (0.126) | 0.086 (0.204) | -0.128 (0.278) | 0.061 (0.260) | 0.054 (0.234) | 0.137 (0.127) | 0.031 (0.206) | -0.185 (0.280) | 0.012 (0.263) | 0.088 (0.232) | 0.125 (0.125) | 0.085 (0.204) | -0.128 (0.278) | 0.065 (0.260) |
| Other | -0.775 (0.423) | 0.457^*^ (0.229) | 0.192 (0.372) | 0.609 (0.509) | 1.135^*^ (0.475) | -0.770 (0.423) | 0.457^*^ (0.230) | 0.194 (0.372) | 0.619 (0.506) | 1.140^*^ (0.476) | -0.775 (0.423) | 0.457^*^ (0.229) | 0.193 (0.372) | 0.609 (0.509) | 1.134^*^ (0.475) |
| Constant | 1.230^**^ (0.194) | 0.896^**^ (0.105) | 1.358^**^ (0.171) | 1.494^**^ (0.233) | 1.228^**^ (0.218) | 1.217^**^ (0.197) | 0.909^**^ (0.107) | 1.310^**^ (0.173) | 1.480^**^ (0.235) | 1.197^**^ (0.221) | 1.235^**^ (0.194) | 0.894^**^ (0.105) | 1.355^**^ (0.171) | 1.496^**^ (0.233) | 1.236^**^ (0.218) |
|  | | | | | | | | | | | | | | | |
| Observations | 442 | 442 | 442 | 442 | 442 | 442 | 442 | 442 | 442 | 442 | 442 | 442 | 442 | 442 | 442 |
| R^2^ | 0.271 | 0.307 | 0.370 | 0.217 | 0.347 | 0.273 | 0.307 | 0.374 | 0.228 | 0.349 | 0.271 | 0.307 | 0.370 | 0.218 | 0.348 |
| Adjusted R^2^ | 0.216 | 0.255 | 0.322 | 0.158 | 0.298 | 0.215 | 0.251 | 0.323 | 0.166 | 0.297 | 0.216 | 0.255 | 0.322 | 0.159 | 0.299 |
| Residual Std. Error | 0.701 (df = 410) | 0.380 (df = 410) | 0.617 (df = 410) | 0.843 (df = 410) | 0.787 (df = 410) | 0.701 (df = 408) | 0.381 (df = 408) | 0.617 (df = 408) | 0.839 (df = 408) | 0.788 (df = 408) | 0.701 (df = 410) | 0.380 (df = 410) | 0.617 (df = 410) | 0.843 (df = 410) | 0.787 (df = 410) |
| F Statistic | 4.917^**^ (df = 31; 410) | 5.857^**^ (df = 31; 410) | 7.752^**^ (df = 31; 410) | 3.673^**^ (df = 31; 410) | 7.030^**^ (df = 31; 410) | 4.654^**^ (df = 33; 408) | 5.485^**^ (df = 33; 408) | 7.380^**^ (df = 33; 408) | 3.659^**^ (df = 33; 408) | 6.634^**^ (df = 33; 408) | 4.910^**^ (df = 31; 410) | 5.868^**^ (df = 31; 410) | 7.762^**^ (df = 31; 410) | 3.682^**^ (df = 31; 410) | 7.062^**^ (df = 31; 410) |
|  | | | | | | | | | | | | | | | |
| Note: | *p<0.05 **p<0.001  Abbreviation: M= mobility, SC=self-care, UA=usual activities, P=pain, AD= anxiety/depression | | | | | | | | | | | | | | |
